# From Pressure to Peril: Investigating the Drivers of Suicide Planning and Attempts in University Students Struggling with Academic Anxiety

**DOI:** 10.3390/bs15121721

**Published:** 2025-12-12

**Authors:** Gulzar H. Shah, Masha Asad Khan, Maham Muzamil, Mahira Ahmed

**Affiliations:** 1 Jiann-Ping Hsu College of Public Health, Georgia Southern University, Statesboro, GA 30460, USA; 2 Department of Psychology, Kinnaird College for Women, Lahore 54000, Pakistan; masha.khan@kinnaird.edu.pk (M.A.K.); mahira.ahmed@kinnaird.edu.pk (M.A.); 3 Department of Education, Kinnaird College for Women, Lahore 54000, Pakistan; maham.muzamil@kinnaird.edu.pk

**Keywords:** suicidal ideation, self-harm, academic anxiety, coping mechanisms, academic pressure, cultural stigma, mental health support, student well-being

## Abstract

Self-harm is a growing public health concern, particularly among university students facing academic anxiety. This study explored the underlying factors driving suicide planning and attempts in this population. Using a qualitative design, we conducted thematic analysis of interviews with eight counselors and eight students from five public and private universities. Analysis via NVivo Software revealed six core themes: (1) current mental health support and coping strategies, (2) triggers of suicidal ideation linked to family and psychological stressors, (3) perceptions of campus mental health services, (4) cultural and societal misconceptions surrounding suicide, (5) institutional barriers to accessing mental health care, and (6) student-driven recommendations for prevention and support. These findings highlight the complex interplay between academic pressure, emotional distress, and limited institutional support. The study emphasizes the need for universities to enhance access to mental health services, reduce stigma through open dialogue and peer support, and engage families through targeted workshops. Tailored interventions addressing academic, emotional, and familial challenges—such as flexible deadlines and improved counseling access—can significantly reduce suicidal ideation and promote student well-being.

## 1. Introduction

Globally, university students’ suicidal tendencies are becoming a serious public health issue. Students are more likely to experience mental health issues, such as suicidal thoughts and behaviors, as a result of the transition from adolescence to adulthood and the academic, social, and personal pressures of university life (Lumivero, 2024; Lipson et al., 2022). The diathesis–stress model provides a valuable framework, suggesting that individual vulnerabilities, such as cognitive biases, impulsivity, or prior mental health issues, interact with environmental stresses like academic demand to increase the risk of suicidal ideation. Suicidal ideation, defined as thoughts of killing or harming oneself, is alarmingly common among the university population and can appear in many forms. These range from brief, passing thoughts to carefully planned intentions (Monroe & Simons, 1991; Landa-Blanco et al., 2024).

Academic pressure is one of the leading causes of mental health difficulties among students. The demanding nature of coursework, frequent examinations, and the pressure to achieve can lead to high levels of stress and anxiety. These factors are closely linked to suicidal ideation (Arria et al., 2021; Eisenberg et al., 2020). Suicidal thoughts and behaviors are prevalent among college students, yet many do not seek help when experiencing a crisis. A survey of over twenty-six thousand undergraduate and graduate students across seventy US colleges found that a substantial proportion reported lifetime or recent suicidal ideation, with academic stress, relationship problems, and emotional distress commonly cited as triggers. Importantly, half of the affected students did not disclose their thoughts or pursue professional support (Drum et al., 2021). This aligns with another research study on student mental health and help-seeking patterns, which found that fifty percent of the students who screened positive for major depressive disorder or anxiety received any mental health services within the past year. Help-seeking behavior was measured as any counseling or psychiatric treatment, and barriers included personal stigma, low perceived need, and limited awareness of services. They found that personal stigma significantly reduced help-seeking, while perceived public stigma showed little impact. Moreover, a substantial treatment gap indicated that many students experiencing mental health issues do not pursue professional help (Hunt & Eisenberg, 2021). A recent 10-year analysis (2007–2017) of 155,026 US college students examined mental health treatment, diagnosis, depressive symptoms, suicidal ideation, and stigma. The study found notable increases in mental health concerns and service utilization alongside a decline in stigma towards seeking help. Despite this improvement, many students with significant symptoms remained untreated. Researchers noted limitations related to self-reported data and survey response bias, highlighting ongoing gaps between mental health needs and service use among students (Jones et al., 2023).

### 1.1. Academic Stress and Students’ Mental Health

Research indicates that students who experience high levels of stress in their academic setting are more susceptible to mental health issues, such as depression and suicidal thoughts (Giancola et al., 2024; Gupta et al., 2024; Gbore & Adebowale, 2023). As they strive for academic success, university students usually struggle with the responsibilities and demands of their educational journey (Gallagher, 2024; T. H. Nguyen et al., 2023; Paredes-Céspedes et al., 2024). On this excursion, some people suffer from extreme academic anxiety, which can set off a domino effect of psychological distress and emotional misery. Given the potential for long-term harm to students’ lives in this setting, the threat of suicide becomes a significant concern (Mann, 2003; Muhammad Khir et al., 2024).

The influence of family dynamics and psychological stressors on students’ mental health is considered a significant factor. According to Wang and Zhang (2024), having family support significantly reduces the risk of mental health problems, but not having family support can worsen psychological discomfort and have serious consequences, including suicidal thoughts and actions. Psychological discomfort and family problems are frequently associated with psychological triggers, such as panic attacks, self-harm, and hopelessness (M. H. Nguyen et al., 2021; Dobbertin et al., 2024; Mortier et al., 2022). As noted by Drum et al. (2021), the prevalence of suicidal ideation among students reflects profound mental suffering, a deep sense of hopelessness, and the perception that they cannot manage academic demands. Students may feel alone and unsupported due to the stigma associated with mental health concerns and the dearth of candid conversations about emotional health (Corrigan et al., 2024; Urme et al., 2022). Cultural settings can influence students’ desire to seek treatment by either helping or impeding the acceptance and support of mental health initiatives (Joiner, 2005).

### 1.2. Aim of the Current Study

In this study, the authors aim to examine how academic distress contributes to suicidal ideation, planning, and attempts among university students. Specifically, it seeks to explore how psychological, familial, cultural, and institutional factors shape students’ experiences with suicidal thoughts and acts. The study also examines how students and university counselors perceive available mental health services, barriers that limit help-seeking, and the kind of support students believe would prevent self-harm. By addressing these questions, the study intends to generate insights that can guide universities in strengthening mental health services.

### 1.3. From Ideation to Attempt

The occurrence of students thinking about suicidal ideation shows the severity of the mental suffering they go through, indicating a deep sense of hopelessness and a belief that they are unable to handle the demands of academic life (Abualhamael et al., 2024). Given this perspective, individuals usually isolate themselves and plan for suicide, which signifies a significant advancement on the spectrum of suicidal ideation, indicating a final turn towards self-harm and maybe ending one’s life (Roberts et al., 2023; Ribeiro et al., 2016; Tang et al., 2024). Suicidal ideation and preparation are essential markers of psychological distress (Diefenbach et al., 2024). Still, the actual act of attempted suicide is a horrific representation of the depths of desperation that college students go through (Heapy et al., 2024; Shaman et al., 2024).

### 1.4. Prevalence and Severity of Suicide Attempts Among University Students

Among full-time college students aged 18–22, about 0.9% reported suicide attempts in the past year compared with approximately 1.9% among adults of the same groups not enrolled in college (Substance Abuse and Mental Health Services Administration, 2024). Among the broader adult population (all ages), the past-year attempt rate is approximately 0.6% (Centers for Disease Control and Prevention, 2025; Corrigan et al., 2024). The act of causing bodily harm to end life, known as an injurious suicide attempt, embodies the severe outcomes of failing to address academic anxiety and untreated mental health issues (da Conceição et al., 2024). The prevalence of harmful suicide attempts among university students needs immediate interventions to reduce the risk factors that lead to such concerning results (Fadipe et al., 2025; Fauk et al., 2023).

### 1.5. Barriers to Accessing Mental Health Services

A crucial concern is that mental health services in academic settings are insufficient or unavailable after the initial session (Guenthner et al., 2023). Students frequently report that counseling services are scarce (Li et al., 2025). Therefore, the majority of students suffer a lot due to this lack of mental health care at the university level (Li et al., 2025). Matar et al. (2024) emphasized that students face several obstacles in receiving mental health treatment, such as the scarcity of counselors, ignorance of available services, and the stigma attached to asking for assistance (Steare et al., 2023). These barriers prevent students from receiving the necessary support, which raises the risk of untreated mental health issues, suicidal thoughts, and actions (Walker et al., 2023).

### 1.6. Limitations of Existing Research

There is an increasing amount of research on suicide ideation and actions among university students. However, there are still many gaps in the precise causes and socio-environmental influences of these problems. Critical characteristics that have been linked to suicidal thoughts in students include sleep quality, social media consumption, self-esteem, and perceived barriers to getting psychiatric treatment (Matar et al., 2024; Mortier et al., 2022). However, rather than delving into students’ complex experiences and perspectives, these studies often focus on broad generalizations. In addition, a significant void in the current body of literature is the dearth of qualitative studies that document the actual experiences of students grappling with suicidal ideation and actions. Gaining insight into the individual experiences and unique stressors that students encounter, especially those associated with academic anxiety, can provide a more comprehensive picture of the issue.

### 1.7. Rationale of Current Study

This study intends to shed light on the underlying causes that drive vulnerable college students from feeling under pressure to the risky verge of planning and attempting suicide by examining the intricate connection between academic distress and suicidal actions. By concentrating on the precise ways that academic demands influence suicidal thoughts and attempts, this study seeks to close this knowledge gap and offer recommendations for improved mental health practices in educational settings. By focusing on how academic pressures influence suicidal ideation and attempts, this research will bridge these gaps to inform customized interventions. The long-term impact will be improved awareness and a lower prevalence of suicidal behaviors in this susceptible group.

## 2. Materials and Methods

### 2.1. Research Paradigm

The present study was guided by a constructivist paradigm, which emphasizes that people create meaning through their individual experiences and social interactions. According to this paradigm, individuals’ interpretations of their lived experiences shape reality, which is seen as subjective and context-dependent (Creswell, 2008). This paradigm supports the study’s goal of examining how people interpret their social and psychological reality in relation to their particular surroundings. To ensure that participants’ viewpoints were understood within their individual and cultural settings. The constructivist orientation guided the study’s design, data collection, and analysis.

### 2.2. Research Design and Sampling

The study employed a qualitative research design to analyze data collected from university students and college counselors through open-ended interview guides. The study focuses on university students and counselors from public and private institutions, and draws a purposive sample of 16 participants, comprising eight counselors and eight students from 3 public and private universities. We limited the sample size to eight because of the nature of the study. Since the study was about the sensitive issue of suicidal thoughts and attempts, it initially posed difficulties in participant recruitment. Several students opted out of interviews, citing concerns about sharing their personal experiences due to worries related to research publication and potential stigma. To address this challenge, we requested that the college campus counselors concerned help connect us with willing participants, ensuring they felt supported and at ease throughout the process. Invitations were sent to students recommended by the counselors. Counselors who provided referrals for students also participated as interviewees. To reduce potential selection bias, we ensured that referrals represented a diverse range of student experiences across disciplines, academic years, and levels of distress.

After completing eight thorough interviews, we reached data saturation. The responses began to reveal common themes, and no new significant information emerged. Acknowledging this repetition, we decided to conclude data collection after eight interviews, as qualitative research aims to gain comprehensive insights and depth rather than focus on a large sample size.

### 2.3. Research Instrument

One research instrument was developed based on open-ended qualitative interview guides. We used two instruments: one for students and one for counselors (Appendix A). The research instrument for students consists of two sections. The first section included questions about students’ demographics, including their gender, age, and discipline. The second section included seven questions about the drivers that influence suicide planning and attempts in university students struggling with academic anxiety. The research tool for counselors consists of seven questions on causes of suicidal planning, its trends and frequency among students, with coping interventions, and recommendations. Diathesis Model (Mann, 2003) and Joiner’s Interpersonal Theory of Suicide (Joiner, 2005) guided the formulation of inquiries regarding perceived burdensomeness, obstructed belongingness, and coping strategies. The tools were pilot tested with two students and two counselors to assess clarity and contextual relevance. This phase proposed minor alterations in phrasing and sequencing to enhance emotional sensitivity and narrative coherence. Each interview lasted approximately 30 min, enabling participants to convey their experiences and insights. Ethical approval for this study was obtained from the Institutional Review Board (IRB) of Kinnaird University, with the protocol ID KC/ORIC/ERC/2025/004.

### 2.4. Data Collection Procedure

Given that suicide attempts and planning are sensitive topics, we collected all of the data using Zoom and WhatsApp calls rather than face-to-face interviews. Participants were informed that their responses would be recorded for transparency and accuracy. For privacy’s sake, participants were instructed to turn off their cameras. All the data were collected from February to March 2025. We conducted a total of 16 interviews, consisting of eight with students and eight with counselors. After that, the responses began to reveal common themes, and no new significant information emerged. Acknowledging this repetition, we decided to conclude data collection after 16 interviews, as qualitative research aims to gain comprehensive insights and depth rather than focus on a large sample size. Informed consent was obtained from all participants in the study, including parental/guardian consent from participants under 18. The checklist of Consolidated Criteria for Reporting Qualitative Research (COREQ) is included in Appendix B.

### 2.5. Data Analysis

We used NVivo software version 15 (Lumivero, 2024) for the thematic analysis of the qualitative data employed to synthesize students’ perspectives of the drivers influencing their academic anxiety and suicide plans and attempts. NVivo facilitated organizing the data, systematic coding, visualization of thematic connections, and identification of cross-group themes and trends, thereby enhancing analytic rigor and transparency. We used three coding passes: open, coaxial, and selective. Open codes were allocated to all potential themes. Using axial coding, specific thematic codes were selected as sub-themes to create a word cloud structure for each more comprehensive subject. Preliminary codes were generated from unprocessed transcripts to facilitate the natural development of themes from participants’ narratives. The transcripts were read multiple times before coding to ensure immersion and familiarity. All transcripts were systematically coded and categorized into themes according to the interview guide. Discrepancies were addressed through consensus, leading to the refinement of the codebook. A separate ID was assigned to each participant to maintain confidentiality and anonymity throughout the study. Non-relevant themes and sub-themes were removed using selective coding to reduce the amount of data.

## 3. Results

Table 1 shows the demographic information of study participants. The study consisted of 16 participants, equally split between students (*n* = 8) and counselors (*n* = 8). The students ranged in age from 18 to 27 (three male, five female); they studied a variety of fields, including psychology, education, law, and the social sciences. The counselors ranged in age from 30 to 55 years and were all female; their professional careers included clinical psychology, educational counseling, and student affairs.

**Figure 1:**
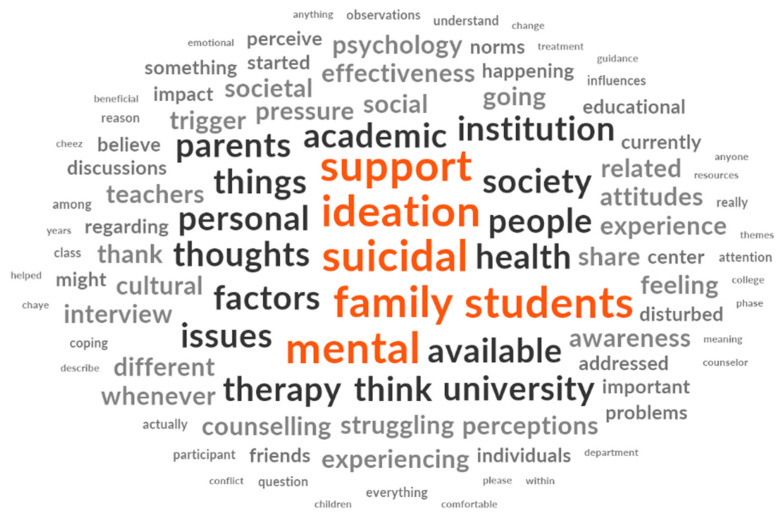
Word cloud representing students’ concerns regarding the drivers of suicide planning and attempts.

The word cloud (Figure 1) depicts the key terms aligning with the constructs from the thematic analysis. For instance, it shows that “support” is a critical factor in preventing or addressing suicidal ideation, as individuals who lack adequate support systems are more likely to experience persistent suicidal impulses. 

Our thematic analysis, discussed later, also revealed that both social support networks and accessible mental health services provide emotional stability, mental health support, and prompt intervention, which serve as protective factors. The term “Family” is also prominent in the word cloud. It is supported by the importance of family influences in suicidal ideation, as suicidal tendencies can be significantly influenced by family dynamics, as specific relationships or parental attitudes can either alleviate or intensify emotional distress, as supported by our thematic analysis. Suicidal thoughts can develop or escalate as a result of family environments that are either unsupportive or strained. “Parents,” “people,” and “mental” are among the most prominent words in our word cloud and thematic analysis.

The themes and subthemes of our analysis are given in the tables below regarding the students’ concerns of suicidal ideation. These themes emphasize the pressing necessity for multi-level support systems and the intricate interplay of personal, social, institutional, and cultural factors that affect students’ mental health. The themes indicate that the students stressed the need for therapy, self-improvement, and awareness programs. The thematic analysis also highlighted that family troubles, stress, lack of support, stigmatizing cultural misconceptions, and pessimism were psychological and emotional triggers for suicidal ideation. The participating students reported confusion, lack of assistance, and cultural stereotypes concerning mental health in academic settings as challenges. Students also indicated that family relationships, societal expectations, and mental health stigma extenuated suicidal ideation. The thematic analysis showed that academic institutions of higher learning, especially private ones, often had inadequate or inaccessible mental health treatments beyond initial sessions. The students suggested the role of workshops, inclusive environments, empathic counselors, and motivating support to prevent suicide and enhance mental health. The subsequent section presents the main themes along with selected quotes.

Theme 1: Current Mental Health Support

Sub-theme: Coping Mechanisms/Effective Coping Strategies

Table 2 represents the subtheme related to coping mechanisms/effective coping strategies. The majority of students shared that they had experienced suicidal ideation a range of times, from a few months to many years. Most students indicated that seeking diverse therapeutic outlets and participating in various activities were essential coping mechanisms.

Theme 2: Triggers of Suicidal Ideation

Sub-theme: Family and Psychological Triggers

Table 3 provides an overview of the subthemes identified in this theme. The majority of students noted that a range of emotional and psychological factors triggered their suicidal thoughts and actions. They expressed emotional suffering, resulting from isolation, withdrawal, and having unsettled mental health issues that were often worsened by institutional and societal expectations. These severe internal conflicts made students feel worthless, and for some, recurring ideation of self-harm.

Theme 3: Perceptions of Campus Mental Health Services 

Sub-theme: Campus Mental Health Services/perceptions/influences

Table 4 presents the core subthemes related to campus mental health services. Several students emphasized their failure to understand the complexity of suicidal situations in the absence of the necessary health education and empathetic attitudes, perceptions, and actions of peers and society. This unfamiliarity may lead to misinterpretation and stigmatization, further isolating individuals who require assistance. Cultural norms and societal beliefs greatly influence attitudes toward mental health, yet these factors are usually disregarded. To promote a more knowledgeable and caring society where people feel empowered to seek support and assistance for mental health issues, this awareness gap must be closed.

Theme 4: Cultural and Societal Impact

Sub-theme: Influences of cultural and Societal factors on Suicidal Ideation

Table 5 shows how participant narratives clustered into cultural and Societal factors on Suicidal Ideation. Many students experience suicidal thoughts for a variety of reasons, including social, family, and educational issues. Overwhelming feelings of hopelessness may result from academic difficulties, such as financial burdens and high performance expectations. Parental attitudes, toxic relationships, and conflicts within the family all have a significant impact on mental health. In addition, shame and isolation among those who are struggling are exacerbated by society and cultural beliefs toward mental health and suicide.

Reflecting on their adverse childhood experiences, some students discussed the subtleties of poor parent–child relationships, unresolved parental disputes, and the nuances of joint family systems that can negatively impact children’s mental health, feeding into suicidal thoughts and necessitating effective therapy options.

A few students shared their struggles with academic pressure, which were complicated by financial difficulties and family pressures. They believed that feeling trapped by these negative influences necessitated social support, and that a lack of it could lead to losing sight of one’s purpose.

Theme 5: Institutional Support and Response to Suicide Ideation

Sub-theme: Mental health support availability in the university/institution

Table 6 presents a summary of themes and subthemes related to the mental health support available in educational institutions. Inadequate mental health care provided by educational institutions is a significant problem in our culture. Many students encounter obstacles in their quest for efficient mental health care, such as stigma, insufficient funding, and ignorance. These difficulties may cause them to feel isolated, anxious, or depressed, which can harm their overall well-being and academic performance. Even while specific programs are offered, their quality and accessibility are often lacking, depriving many students of the support they need. It is essential to close these gaps to create a nurturing atmosphere where children can flourish intellectually and personally. It is time for our educational systems to prioritize mental health.

Theme 6: Recommendations for Support and Prevention

Sub-theme: Student-Driven Suggestions and Solutions

Table 7 provides participant quotes on suggestions and solutions. Many students reported that there are insufficient awareness programs and support systems on campus. To prevent suicidal ideation, educational institutions should provide a supportive environment and a sufficient resource system to prevent suicidal commitments.

Services: The following tables (Table 8, Table 9, Table 10, Table 11, Table 12, Table 13 and Table 14) present the themes and sub-themes, with selected quotes emanating from counselors’ perceptions. Their interviews provided insights into student suicide ideation prevalence, causes, and institutional reactions. They observed persistent discomfort stemming from academic, familial, and cultural demands, as well as inadequacies in the major support infrastructure. The following themes capture a sophisticated understanding of these difficulties and evidence-based strategies to improve school mental health support systems.

## 4. Discussion

### 4.1. Summary of Findings

This qualitative study investigated perspectives of university students and counselors about the drivers of students’ suicidal ideation and attempts, especially those resulting from academic distress. Interviews were conducted with eight students and eight counselors from five public and private universities. This study’s primary objective was to investigate how academic pressures contribute to suicidal ideation and attempts among university students. Several themes emerged from qualitative data from students and counselors. Both groups emphasized the critical role of academic pressure, family expectations, and lack of emotional support in triggering suicidal ideation. Furthermore, participants agreed on the value of reducing mental health stigma and enhancing the availability of timely and compassionate institutional support services. Both students and counselors emphasized the interplay between intense academic demands, limited emotional, and insufficient institutional resources as critical pathways leading to suicidal ideation. The findings highlight the urgent need for culturally sensitive mental health interventions that address both individual vulnerabilities and environmental stressors.

### 4.2. Thematic Discussion

#### 4.2.1. Current Mental Health Support and Coping Strategies

The results show that students face severe emotional distress and frequently feel overwhelmed to the point of contemplating withdrawal because of the demanding academic schedule. Family dynamics, societal views, and the accessibility of mental health resources significantly shape students’ mental health status. These findings highlight the necessity of all-encompassing mental health interventions in school settings to address the complex factors contributing to suicidal behaviors among students. Students’ experiences of severe emotional distress, including suicidal thoughts and behaviors, align with previous studies highlighting the prevalence of mental health problems among young adults. For instance, Landa-Blanco et al. (2024) found that university students are particularly vulnerable to suicidal thoughts and actions because of the significant academic and social pressures they encounter. This is in line with our study findings, indicating that students frequently struggle with intense internal distress that pushes them to emotional collapse, particularly if they lack effective coping mechanisms. Furthermore, their emotional immaturity and lack of life experience may make their mental health issues worse.

Our findings add to previous research, providing a culturally sensitive viewpoint on the elements influencing students’ mental health issues. Hetrick and Sharma (2025) noted that counselors often come across students contemplating suicide during stressful academic times, especially final-year students who are feeling overburdened by expectations from their families and educational failure. In contrast, counselors in our study emphasized culturally specific triggers, such as forced career paths, authoritarian parenting, and social pressures related to honor and reputation. These issues are less stressed in Western research but are highly relevant in South Asian contexts (Alshareef & Alfuqaha, 2024). Additionally, while other research has shown financial stress as a significant contributing factor (Yao et al., 2023), the counselors participating in our study underscored relational trauma and emotional neglect as more pressing concerns.

#### 4.2.2. Triggers of Suicidal Ideation Linked to Family and Psychological Stressors

The influence of family dynamics and psychological stressors on the mental health of students is significant. Our findings suggest that psychological distress and family problems are linked with panic episodes, self-harm, and hopelessness. Research from Makhubela (2021) demonstrates that family support is the main factor against mental health difficulties in university students. On the other hand, a lack of family support can make psychological anguish worse, which can have severe consequences like suicidal thoughts and actions. One additional important feature in your findings is the general lack of understanding regarding suicide issues among students and the community at large. Numerous students have never participated in mental health lectures or workshops, indicating inadequate mental health education. Another study by Mortier et al. (2022) highlights the need for greater mental health awareness and education to avoid suicidal attempts. The lack of these programs can make kids more susceptible to suicidal planning since they are ill-prepared to deal with their mental health issues.

Most study participants reported that societal and cultural expectations have a significant impact on their mental health. Students often experience feelings of loneliness and lack of support due to the stigma related to mental health concerns and the absence of candid conversations about emotional well-being. This is consistent with findings from Corrigan et al. (2024), who contend that societal stigma is a significant obstacle to getting mental health treatment. The acceptance and support of mental health efforts can be influenced by cultural context, which can either positively or negatively affect students’ desire to seek help. Students who are under academic pressure, have personal and familial difficulties, and lack emotional support are among the many variables that lead to suicidal planning. Our results are consistent with previous research, like that of Lipson et al. (2022), who found that personal problems and academic stress are significant predictors of suicidal thoughts in college students. These problems are exacerbated by limited access to mental health resources and effective counseling (Andrew et al., 2011; Yorgason et al., 2024).

#### 4.2.3. Perception of Campus Mental Health Services

A critical theme that emerged in our findings was institutional deficiencies in addressing the issue of suicidal ideation, in addition to individual and cultural influences. Fadipe et al. (2025) and Fauk et al. (2023) highlighted a persistent gap between institutional best practices and the mental health treatments currently offered in educational institutions. While some colleges provide basic counseling services, their effectiveness is compromised by a shortage of qualified personnel, outdated evaluation methods, inadequate privacy, and limited infrastructure. The counselors noted that weak institutional support and fragmented referral networks were issues that mirrored global trends but were exacerbated by regional limitations. On the other hand, some components, such as the use of cognitive behavioral therapy and spiritual counseling, were considered culturally congruent. However, long-term progress is still impeded by uneven training and administrative carelessness. Compared to Western institutions with formalized mental health frameworks, Pakistani universities continue to fall short of properly prioritizing psychological care, reinforcing systemic neglect (Asad & Dawood, 2025).

#### 4.2.4. Cultural and Societal Misconceptions Surrounding Suicide

A pervasive cultural stigma surrounding mental health is considered a significant contributory factor to suicidal ideation, where social judgment and victim-shaming prevent students from seeking mental health counseling and care (Rahman et al., 2025; Shah & Alamian, 2023). Although awareness and openness have improved over the past decade, students still tend to worry about social stigma and adverse reactions (Karnwal & Sharmila, 2024; Zhai et al., 2025).

#### 4.2.5. Institutional Barriers to Accessing Mental Health Care

One crucial concern raised by our findings is the accessibility of mental health services in academic settings. Numerous students complain about the shortage of counseling services, and those that are offered are sometimes inadequate or inaccessible beyond the first few sessions. Studies such as Gallagher (2024) support this finding, showing that, although many colleges provide counseling services, they are often underfunded and unable to meet demand. Students may not have the tools they need to properly manage their mental health because of this gap in mental health care. Furthermore, accessibility issues with mental health services are another critical element of our research. Students encounter numerous challenges, including the scarcity of counselors, limited awareness of available services, and the stigma associated with seeking help. This aligns with research in the literature, where studies such as Zhou et al. (2023) highlight comparable obstacles that prevent students from accessing mental health services. These obstacles raise the possibility of suicidal planning and attempts by contributing to untreated mental health problems.

#### 4.2.6. Student-Driven Recommendation for Prevention and Support

Our study findings are consistent with existing literature on student suicidal planning and attempts. Several important variables that affect students’ mental health include themes of emotional turmoil, family dynamics, societal attitudes, and institutional support. Educational institutions must address these factors to ensure students have access to appropriate mental health resources and support. Institutions can promote general well-being and prevent suicidal tendencies among students by recognizing and addressing these variables. This entails raising family support levels, decreasing the stigma associated with mental illness, raising mental health awareness, and expanding the availability and accessibility of mental health treatments in university settings.

### 4.3. Limitations of the Study

Our study used a qualitative research design to explore university students’ and counselors’ perceptions about suicidal ideation, resulting in study limitations with implications for the generalizability of our findings. First, the study included only 16 individuals from five universities, which may not fully represent Pakistan’s diverse experiences and opinions across regions, disciplines, and institutions. Secondly, suicidal ideation and mental health are sensitive; therefore, students and counselors may have suppressed information or altered their opinions to fit societal norms. Third, the study only captured individuals’ experiences and perceptions at one point in time, making it difficult to determine how suicidal ideation or support mechanisms change. Fourth, the findings rely exclusively on interviews, without including institutional data (e.g., the number of reported mental health cases and resource allocation), which could have strengthened the analysis and validation of observed trends. Fifth, the study did not use any standardized clinical assessments of depression or personality traits to select participants. While participants were included based on prior behavioral health service use, the lack of standardized screening for personality or behavioral disorders limits the ability to contextualize their experiences within established diagnostic frameworks. Finally, the qualitative interviews yield context-specific results. They are not statistically applicable to Pakistani or international students.

### 4.4. Future Directions and Implications

This qualitative study provides avenues for developing theory in suicidal ideation, while contributing to the development of culturally relevant interventions. The qualitative nature of the study has highlighted the complexity of an individual regarding the major mental health issue of suicidal ideation. By revealing culturally ingrained interpretations of distress and suicide, qualitative research contributes to the formulation of interventions that align with individuals’ lived experiences (Richardson et al., 2025; Amiri et al., 2025). Such alignment ensures that interventions and strategies tackle not only psychological risk factors but also sociocultural dynamics that affect help-seeking and coping mechanisms. Gangemi et al. (2019, 2021) explain how reasoning strategies and emotional reasoning processes worsen cognitive misrepresentations such as despair and entrapment, which are pivotal to suicidal ideation. These insights suggest that future interventions should incorporate cognitive–behavioral elements to help individuals restructure their maladaptive reasoning patterns. Qualitative studies can provide further insight into how an individual forms their perception of the world around them through relationships and cultural norms. Future qualitative research aimed to provide research evidence to inform suicide prevention efforts could also use tested theoretical frameworks, such as Rasmussen et al.’s (2025) integrated Motivational/Volitional model of suicide prevention and treatment, and suggest that, in addition to addressing the motivational states associated with self-stifling and humiliation, in order to prevent progression to suicidal behaviors. In sum, future research may build on these findings and generate additional research evidence to integrate qualitative narratives into clinical practice, enabling therapists to respond to the subjective meanings individuals assign to their distress.

The following recommendations, based on the study findings, can inform programs aimed at addressing the issue of suicidal behaviors among university students: To begin with, the educational institutions can introduce structured teacher training programs and capacity-building workshops aimed at enhancing teachers’ ability to recognize early indicators of psychological distress among students, such as sudden mood changes, withdrawal from peers, or academic decline. In addition, teachers should be guided on the referral mechanism to connect at-risk students with school counsellors or psychological support units. Furthermore, schools can integrate brief, structured mental well-being activities into daily routines or before high-stress events, such as examinations. These may include mindfulness and breathing exercises, simplified cognitive behavioral techniques (CBT), and Emotion regulation exercises. To extend these efforts beyond the school setting and to reinforce school-based efforts, a community-based awareness campaign can be organized in collaboration with local NGOs and psychologists. These initiatives can train peer facilitators to support your mental health, encourage open discussions on emotional well-being, and provide information on accessible counselling resources in both urban and rural settings.

## 5. Conclusions

The results of this study revealed the influence of different drivers, such as academic pressure, family distress, and emotional misery, on suicidal planning and suicidal thoughts among university students, as well as the intense mental distress they go through. Without healthy coping strategies, students frequently experience inner demons that push them to the verge of despair, which can result in self-destructive behavior and suicidal thoughts. Families are a significant source of stress for many people with mental health disorders, and mental health interventions must take family dynamics into account. The problem is worsened due to a noticeable ignorance about suicide issues and students’ inability to identify the warning signs of mental health issues, which delays seeking assistance and intervention.

Societal and cultural beliefs also greatly influence students’ mental health. Students often experience feelings of loneliness and lack of support due to the stigma associated with mental health concerns and the absence of candid conversations about emotional well-being. This social stigma may discourage students from getting the necessary support, worsening their mental health issues. To provide a setting where students feel comfortable and encouraged to talk about their mental health concerns, these societal and cultural barriers must be addressed. Students may experience significant levels of stress and anxiety as a result of the demanding academic requirements, and these conditions are intimately linked to suicidal ideation. These problems are worsened by the lack of adequate mental health resources and counseling in educational institutions, which deprives students of the assistance they need to manage their mental health properly. A comprehensive, multi-level strategy is necessary to address the issues highlighted by our findings. By increasing services, lowering stigma, and creating supportive environments, educational institutions must put mental health first. Universities can foster a culture of care that supports student well-being and reduces the incidence of suicidal behaviors by incorporating mental health education, involving families, and enhancing institutional infrastructure.

## Figures and Tables

**Table 1:** Participants’ Demographics information.

Group	Number (*n*)	Age Range	Gender	Academic/Professional Background
Students	8	18–27 years	3 Males, 5 females	Psychology, Education, Law, Social Sciences
Counselors	8	30–55 years	All female	Clinical Psychology, Educational Guidance, Student Affairs

**Table 2:** Description of the themes and subthemes used for the interviews conducted with undergraduate students regarding Current Mental Health Support.

Major Themes	Subthemes	Descriptions	Selected Quotes
Current Mental Health Support	Coping Mechanisms/Effective Coping Strategies	**Coping Mechanisms/Effective Coping Strategies:** Techniques and methods individuals use to manage and reduce suicidal ideation. **Personal Improvement:** Personal growth and changes that help mitigate suicidal thoughts. **Therapy is Important:** The role of therapeutic interventions in preventing and managing suicidal ideation. **Awareness Programs:** The impact of programs aimed at raising awareness about suicidal ideation and prevention.	Here is what different students said: I’ve experimented with various therapeutic techniques and kept myself occupied with calligraphy and photography. Depending on how severe the problem is, different exercises are performed. Academic webinars and seminars help people understand the value of different treatments. One book altered my perspective, and I gained a deeper understanding of life’s purpose through a class assignment. I used to think that killing myself would make me happy, but then I saw that I had to accomplish something positive for both my life and the lives of others. In my experience, discovering joy in minor accomplishments can be a potent barrier against hopelessness. I use constructive coping mechanisms daily that help me turn my gloomy moments into learning experiences. I prefer to seek assistance from others is a brave first step on the road to recovery and fortitude. Other respondents said: Participating in joyful activities helps me stay anchored during stressful moments. Creating a network of dependable people is a lifesaver when things go tough. Self-care and mindfulness exercises build a wall against overpowering ideas. Making a safety plan with people you can trust gives you hope and security. Concentrating on future goals and aspirations facilitates redirecting my negative thoughts toward positive outcomes.

**Table 3:** Description of the themes and subthemes used for the interviews conducted with undergraduate students regarding Triggers of Suicidal Ideation.

Major Themes	Subthemes	Descriptions	Selected Quotes
Triggers of Suicidal Ideation	Family and Psychological Triggers	**Family-related Factor Triggers:** How family dynamics and issues can initiate or exacerbate suicidal thoughts and behaviors. **Academic Stressors:** The influence of academic pressures and stress on suicidal ideation. **Lack of Support System:** The effect of not having a supportive network on individuals experiencing suicidal thoughts. **Pessimistic Attitude/Negative Attitude Towards Life:** How negative thinking patterns contribute to suicidal behaviors. **Hopelessness:** The role of feelings of hopelessness in suicidal ideation. **Pressures:** Various pressures that contribute to suicidal thoughts. **Panic Attacks During Suicidal Ideation:** The occurrence of panic attacks alongside suicidal thoughts.	Some representative quotes are listed below: I experienced a constant sense of deprivation and criticism for my beliefs. Psychologists stated I was fine except for being agitated. Teachers’ disregard for their students resulted in poor self-worth and worthlessness. My in-laws’ thoughts caused panic attacks, which led to self-harm. I felt alone because of my introverted personality and my inability to handle constant pressure; devoid of enthusiasm for studying and despondent about finishing my degree or thesis. Emotional distress frequently drives me to the verge of hopelessness, where psychological factors trigger me to set off a tempest of suicidal ideas, leaving me as a victim who feels helpless. I feel an ongoing struggle with inner demons that set me on a hazardous route toward self-destruction due to unresolved emotional suffering. I feel incessant attacks of the mind might cause me to contemplate the impossibly extreme, resulting in detrimental manifestations of psychological discomfort and thoughts of suicide.

**Table 4:** Description of the themes and subthemes used for the interviews conducted with undergraduate students regarding Perceptions of Campus Mental Health Services.

Major Themes	Subthemes	Descriptions	Selected Quotes
Perceptions of Campus Mental Health Services	Campus Mental Health Services/perceptions/influences	Campus Mental Health Services How campus Mental Health Services impacts the recognition and management of suicidal ideation. Lack of Awareness: There is a general lack of understanding about suicidal issues within the community. Lack of Adequate Support/Support System: Insufficient support systems for individuals experiencing suicidal thoughts. External Factors: Outside influences that affect suicidal ideation. Ignore Internal Factors: Neglecting personal, internal factors that contribute to suicidal thoughts.	Here is what different students said: I am accused of my behavior. The teacher in our department made fun of my problems. I face criticism for my deeds, but nobody acknowledges the suffering they caused. People treat suicidal thoughts as attention-seeking, dismissing their actuality. Society frequently dismisses those who exhibit suicidal thoughts and behaviors as mentally ill, neglecting their underlying issues. People make fun of such suicidal ideation concepts. Society started giving the excuse that if a person shows certain behaviors related to suicide, it might be because of a mental illness. Whenever I showed suicidal behaviors or whenever I got panic attacks, everyone made fun of me as if I was pretending or something. I was ridiculed when I showed my distress, as if my suffering was a show. People did not hear my pleas for assistance; they thought my panic attacks were dramatic.

**Table 5:** Description of the themes and subthemes used for the interviews conducted with undergraduate students regarding Perceptions of Cultural and Societal Impact.

Major Themes	Subthemes	Descriptions	Selected Quotes
Cultural and Societal Impact	Influences of cultural and Societal factors on Suicidal Ideation	**Academic Stressors:** The pressure and stress from academic responsibilities that lead to suicidal thoughts. **Family Relationships/Parental Attitude:** The impact of family dynamics and parental attitudes on suicidal ideation. **Family-related Issues:** Specific issues within the family that contribute to suicidal thoughts. **Society and Culture Attitude:** The broader societal and cultural attitudes towards suicide and mental health. **Social Support/Support System:** The importance of having a strong support network in mitigating suicidal thoughts. **Treatment Suggestions:** Recommendations for treatments to manage and reduce suicidal ideation. **Personal Improvement/Realization:** How personal growth and self-awareness can help control suicidal thoughts.	Here are the responses from different students: I am demoralized by my parents’ ongoing arguments. I genuinely know people who had suicidal thoughts because of their parents. Parent workshops are also necessary. Suicidal thoughts, in my opinion, stem from familial problems. I believe that masculine dominance permeates both my family and our society. I find societal conventions disturbing, particularly the notion that girls should be married by the time they are 20 or 22. I feel better when I talk to someone about my issues. Treatment, in my opinion, ought to be based on how serious suicidal thoughts are. After discussing, if my problems are fixed, I feel at ease. To console myself, I have to work hard. I know it is due to family matters; others also know it. I have known individuals who have suicidal thoughts because of their parents. Others said: Suicidal thoughts, in my opinion, are a result of unresolved parental disputes and arguments. Suicidal thoughts may arise from a bad parent–child connection. The mental health of their children is greatly influenced by the way parents live together and how they parent. Disturbances might also result from family disputes. Joint family systems, in my opinion, involve a variety of elements that can impact kids’ mental health. In a shared family arrangement, elders are unable to provide each child with the individual attention they need because there are many of them. Therapy conducted in person is prohibited in joint family systems. I believe it is essential to have a range of therapy options. The ineffectiveness of online therapy can be attributed to improper interactions between psychologists. Individuals going through difficult times or those who have suicidal thoughts need to know that they are not alone. A few students shared that: I had to retake the entire semester because I was unable to administer my final exams. I believe there are not many people who could offer you guidance. Supportive people surround me. My buddies helped to create a network of social support. I think that listening to someone can be therapeutic. During the intermediate, my relatives put pressure on me. My background was middle-class, and the academy was expensive. My relatives used to rage at me and accuse me of not getting the grades they wanted. For me, the combination of these elements created a loop. I believe that when something is removed from its intended purpose, people can become lost.

**Table 6:** Description of the themes and subthemes used for the interviews conducted with undergraduate students regarding Institutional Support and Response to Suicide Ideation.

Major Themes	Subthemes	Descriptions	Selected Quotes
Institutional Support and Response to Suicide Ideation	Mental health support availability in the university/institution	Mental Health Support Availability in University/Institution: The availability and effectiveness of mental health services within educational institutions. Mental Health Support Availability in Private Universities/Institutions: Access to mental health services in private universities and institutions. Lack of Effective Counseling Services: The insufficiency of counseling services to address suicidal ideation effectively.	Most of the students shared that: Despite being an excellent student, I was unable to attend lectures on mental health awareness at my university due to the absence of a counseling committee. Organizations ought to create these committees to promote the psychological health of their students. Teachers frequently do not consider the personal or family difficulties that students may encounter. While some older educators may offer empathy, it is often ineffective to raise issues related to missed deadlines; in fact, some educators may make light of them. Not many counselors are available, and the ones who are may not remain so after the initial session. Although webinars and seminars aim to raise awareness, receiving assistance from friends and teachers depends on emotional expression. Despite its high cost, my academy did not address the mental health concerns of its students. I do not think any educator has ever asked why something was occurring. Although there is still a long way to go, private colleges are beginning to take their students’ mental health and well-being into account.

**Table 7:** Description of the themes and subthemes used for the interviews conducted with undergraduate students regarding “Recommendations for Support and Prevention”.

Major Themes	Subthemes	Descriptions	Selected Quotes
Recommendations for Support and Prevention	Student-Driven Suggestions and Solutions	Conduct seminars/workshops: Conducting seminars/workshops for those who do not want to share their feelings with anyone. Supporting/Inclusive environment in educational institutions Provision of a supportive/Inclusive environment in educational institutions. Motivational talk on general topics during class. Teacher motivational talk, constructive feedback, and one-on-one meetings with those who find studying difficult. Cooperative behavior of the campus counselor Campus counselor positive behavior that allows the patient to discuss/share thoughts without judgment.	Most students stated that: Awareness programs, support systems, and family counseling should be made available to address these issues. They further elaborated, saying that a counselor who is more empathetic towards you, listens to you, and can offer you comfort and solace in a particular environment. It will provide a supportive environment for counselees to express their concerns regarding why they want to commit suicide. A few students reported that: They tried reading motivational books that have changed their perspective. They further said that people sometimes get lost because their lives lack meaning, and they find it difficult to solve the problems they face. Additionally, doing some exercise also helped them to divert their attention from the suicidal planning and attempts.

**Table 8:** Description of the themes and subthemes used for the interviews conducted with counsellors regarding Prevalence and Patterns of Suicidal Ideation.

Major Themes	Sub-Themes	Description	Selected Quotes
Prevalence and Patterns of Suicidal Ideation	Frequency of Encounter and Observed Trends	Investigates the frequency with which experts come across pupils who are planning suicide and focuses on recurring trends among impacted students who make suicidal attempts, or triggers.	The majority of counselors stated that: They frequently meet students with suicidal ideation, especially during exam periods, after the exam results are announced, and relationship breakups. “Forced study” by the parents, i.e., the student wanted to study medicine but was sent by the parents to an engineering university. The number of such cases is not so many, but in every semester, 3–4 students come with this type of thought. Especially students from the 7th semester come with suicidal ideation because they have spent a long time at university with poor grades and even never tell their parents, especially residents of hostels, as they are away from home. Still, when the last year is finished, parents will ask, “When will you start your job to help us?” This fear pushes them to think about suicide. A few counselors highlighted that: The typical patterns/trends of social ideation include family issues, neglected child, history of sexual abuse, absence of parents, unpleasant home environment, academic stress, bullying, pressure from parents, peer pressure, low self-esteem, social media pressure, and underlying mental health disorders.

**Table 9:** Description of the themes and subthemes used for the interviews conducted with counsellors regarding Contributing Factors to Suicidal Ideation.

Major Themes	Sub-themes	Description	Selected Quotes
Contributing Factors to Suicidal Ideation	Social and Family Factors and Academic Stressors	Examines how parental pressure, family conflict, and academic failure trigger suicidal ideation.	Most of the counselors shared that: Most of the social factors include loneliness, homesickness, hostility, and peer pressure. Strict parenting, Lack of emotional support, domestic violence, Lack of trust, Low self-esteem, Peer pressure, Substance abuse, Identity crisis, bullying, Body-shaming, Sexual abuse, and Emotional abuse are common issues that lead to severe mental health issues and result in suicide. Family-related issues are mainly linked with the comparisons within or between families, financial problems, and relationship issues that include parental quarrels and cross-gender friendships. Others reported that the most frequent academic challenges include fear of failure, heavy course loads, poor time management, and extreme competition, which are common among engineering students. A few stated that: Some students feel shame when they cannot meet their own set goals, start to blame themselves, overthink about unprecedented outcomes, and commit suicide.

**Table 10:** Description of the themes and subthemes used for the interviews conducted with counsellors regarding the Institutional Support and Mental Health Services.

Major Themes	Sub-Themes	Description	Selected Quotes
Institutional Support and Mental Health Services	Availability of Resources and Effectiveness of Support Services	Evaluates the availability and use of on-campus counseling and mental health services. Moreover, assess the quality and effectiveness of the available psychological support services.	Many of the counseling staff reported that: Relevant resources are available, and faculty members are also provided with basic training. They said they have expert doctors and psychologists in their educational institution who deal with students suffering from mental health issues. Others reported that: Various relevant interventions and strategies are employed, including Cognitive Behavioral Therapy (CBT) for emotional regulation. Reported cases are given proper treatment until they recover fully; alternatively, severe cases are hospitalized for proper medication and treatment.

**Table 11:** Description of the themes and subthemes used for the interviews conducted with counsellors regarding Cultural and Societal Influences on Help-Seeking.

Major Themes	Sub-Themes	Description	Selected Quotes
Cultural and Societal Influences on Help-Seeking	Cultural Norms and Stigma	Explains how students’ desire to ask for aid is impacted by cultural ideas, stigma, or fear of being judged.	Many of the counselors stated that: In Pakistani society, we do not address the problem at its initial level but instead wait for it to worsen. Some parents discourage female students from seeing a counselor who is struggling with finding a life partner, believing it affects their social image. This shame-based thinking keeps many students from reaching out until a crisis has deteriorated. Others said that: If mental health is stigmatized or seen as a sign of weakness, students may feel ashamed or fearful of being judged, which discourages them from reaching out. Conversely, in environments that promote openness, empathy, and emotional well-being, students are more likely to seek support early.

**Table 12:** Description of the themes and subthemes used for the interviews conducted with counsellors regarding Institutional Challenges in Providing Support.

Major Themes	Sub-Themes	Description	Selected Quotes
Institutional Challenges in Providing Support	Internal Barriers	Identify institutional limitations, including shortages of qualified personnel, financial constraints, and policy inconsistencies.	Many counselors said that: The most significant obstacle is Low funding: we lack sufficient funds for awareness campaigns, workshops, and online resources related to mental health services. A few reported that: There is insufficient staff in one counseling center. Only one or two counselors cannot serve thousands of students. Additionally, 24/7 Availability is necessary on campus for Hostilities students who frequently experience emergencies on weekends or at night when services are unavailable. Others said we need properly trained hostel Resident Tutors (RTs) who provide services when the counselor is off duty.

**Table 13:** Description of the themes and subthemes used for the interviews conducted with counsellors regarding Effective Interventions and Strategies.

Major Themes	Sub-Themes	Description	Selected Quotes
Effective Interventions and Strategies	Successful Approaches	Offer pupils professional advice on interventions that have yielded favorable results.	Most of the counselors said: It is difficult to specify one intervention or support strategy because each case is different when addressing suicidal ideation. They said it is essential first to identify the trigger and urge behind the suicidal ideation systematically by providing emotional support and creating an atmosphere of love and care around the counselee. Others suggested that various interventions can be applied, including psychoeducation, active listening, Cognitive Behavioral Therapy (CBT), Rational Emotive Behavior Therapy (REBT), Acceptance and Commitment Therapy (ACT), Non-judgmental presence, and Peer support programs. A few stated that: Incorporating Mindfulness and self-regulation, as well as religious coping techniques, is also very useful, depending on the nature of the case. During this process, collaborating with psychiatrists is crucial for cases that require medication or intensive therapy.

**Table 14:** Description of the themes and subthemes used for the interviews conducted with counsellors regarding Recommendations for Improvement.

Major Themes	Sub-Themes	Description	Selected Quotes
Recommendations for Improvement	Service Enhancement	Provide suggestions for enhancing or growing the university’s outreach and mental health services.	Most of the counselors recommended that: Different suicide prevention campaigns, awareness campaigns, and Emergency contacts for support should be conducted. These awareness campaigns should be conducted for all faculty members, students, parents, and the community, including college staff members, by highlighting the signs and severe outcomes of mentally disturbed individuals. A few reported that: There should be an increase in the budget for mental health services. Hiring sufficient staff, such as counselors and psychiatrists, is crucial in educational institutions to meet the growing demands of an increasing number of students enrolled.

## Data Availability

The institutional data protection policies of Kinnaird College for Women restrict public access to individual-level qualitative data due to the sensitive nature of the topic, behavioral health issues, and the risk of participant re-identification. De-identified transcripts and materials supporting the findings of this study may be made available upon reasonable request under a data use agreement subject to approval by the Kinnaird College Institutional Review Board and Data Access Committee. Data requests can be emailed to masha.khan@kinnaird.edu.pk with a statement of research purpose, assurance of confidentiality and ethical use, and institutional ethics committee approval.

## Appendix A

Research Tools:

From Pressure to Peril: Investigating the Drivers of Suicidal Ideation and Attempts in University Students

Questions Guide for Counselors

Instructions:

Thank you for your participation. This study aims to understand the social, family-related, and academic factors contributing to suicidal ideation and attempts in university students. The interview will take approximately 30 min. Your responses are confidential and anonymous. You may skip any question or stop at any time.

1. How often do you encounter students experiencing suicidal ideation, and are there any common patterns or trends you’ve observed?
2. What factors do you believe are most significant in triggering suicidal thoughts among students? *Possible probes: Social factors, family-related issues, and academic pressures.
3. How do you evaluate the effectiveness of the mental health resources currently available to students with suicidal ideation at the Guidance and Counseling Center?
4. In your opinion, how do societal norms and cultural attitudes impact students’ willingness to seek help for mental health concerns?
5. What barriers within the institution limit your ability to provide adequate support for students struggling with suicidal ideation?
6. Based on your professional expertise, what interventions or support strategies have you found to be most successful in addressing suicidal ideation?
7. What improvements or changes do you suggest for enhancing mental health services for students experiencing suicidal thoughts?

From Pressure to Peril: Investigating the Drivers of Suicidal Ideation and Attempts in University Students

Questions Guide for Students

Instructions:

Thank you for your participation. This study aims to understand the social, family-related, and academic factors contributing to suicidal ideation and attempts in university students. The interview will take approximately 30 min. Your responses are confidential and anonymous. You may skip any question or stop at any time.

1. For how long have you been experiencing suicidal ideation?
2. Are you currently receiving any therapy or support? If yes, please specify.
3. Please share your thoughts on what type of social, family-related, or academic factors trigger your suicide ideation.
  - *Probe: If a student fails to mention any of the social, family-related, or academic factors triggers, ask for the follow-up example (1-A). So you have talked about the social factors, but what are academic factors, such as homework workload, class environment, etc., that contribute to suicide ideation?
4. How do you perceive the availability and effectiveness of mental health resources available at Guidance and Counseling Center for students experiencing suicidal thoughts?
5. In what ways do you think cultural attitudes and societal norms in our society impact discussions and perceptions of suicide and mental health?
6. Could you describe any personal experiences or observations regarding how suicide ideation is addressed within our educational institution?
7. What kind of support or treatment do you believe would be most beneficial for individuals struggling with thoughts of suicide?

## Appendix B

COREQ checklist

From Pressure to Peril: Investigating the Drivers of Suicidal Ideation and Attempts in University Students

Domain 1: Research Team and Reflexivity

**Item**	**Description**
Interviewer/facilitator	Prof. Dr. Gulzar Shah, Prof. Dr. Masha Asad Khan, Ms. Maham Muzamil, and Ms. Mahira Ahmed
Credentials	Department Chair & Professor, Academic Dean & Professor, Lecturer, Assistant Professor.
Experience and training	Experienced qualitative researchers and educators
Relationship established	No prior relationship with participants
Participant’s knowledge of the interviewer	Participants were informed of the researchers’ institutional affiliation and study purpose
Interviewer characteristics	Researchers maintained neutrality and followed IRB-approved ethical protocols

Domain 2: Study Design

**Item**	**Description**
Methodological approach	Thematic analysis
Theoretical framework	Interpersonal Theory of Suicide (Joiner, 2005); Stress-Diathesis Model (Mann, 2003)
Participant selection	Purposive sampling
How participants were approached	Via institutional channels and direct invitation
Sample size	16 total (8 students, 8 counselors)
Non-participation	3 reported
Setting of data collection	Remote interviews via Zoom and WhatsApp
Description of sample	University students and counselors from different Universities in Lahore City, Punjab, Pakistan.
Interview guide	Developed from literature; pilot tested and refined
Repeat interviews	No
Audio/visual recording	Yes
Field notes	Reflexivity journals and analytic memos are maintained
Duration	Average interview duration: 30 min
Data saturation	Yes, discussed and achieved
Transcripts returned	Yes, transcripts and findings were shared with participants for validation

Domain 3: Analysis and Findings

**Item**	**Description**
Number of data coders	Two independent coders for reliability checks
Description of coding tree	Codes derived inductively and clustered into themes
Derivation of themes	Themes emerged from data; not pre-identified
Software used	NVivo 14 for coding and thematic analysis
Participant checking	Yes, findings reviewed by participants
Quotations presented	Yes, anonymized quotations are used to support themes
Data and findings are consistent	Yes, findings grounded in participant narratives
Clarity of major/minor themes	Major and minor themes are clearly distinguished and discussed
